# Decomposing multidimensional child poverty and its drivers in the Mouhoun region of Burkina Faso, West Africa

**DOI:** 10.1186/s12889-020-8254-3

**Published:** 2020-01-31

**Authors:** Cynthia L. Fonta, Thomas B. Yameogo, Halidou Tinto, Tiff van Huysen, Hamtandi Magloire Natama, Adelaide Compaore, William M. Fonta

**Affiliations:** 1 L’instut de La Recherche en Science de La Sante, l’Unité de Recherche Clinique sise à Nanoro (l’URCN), Ouagadougou, Burkina Faso; 2 School for Policy Studies, University of Bristol, 8 Priory Road, Bristol, BS8 1TZ UK; 3 West African Science Service Center on Climate Change and Adapted Land Use, Competence Center, Blvd Mouammar Kadhafi, Ouagadougou, 06 BP 9507 Burkina Faso; 4 The Earth Institute, Columbia University in the City of New York, New York, NY USA; 5 Climate Change and Green Growth Department (PECG), African Development Bank, Abidjan, Côte d’Ivoire

**Keywords:** Burkina Faso, Multidimensional child poverty, Deprivations, Poverty decomposition, Alkire and Foster methodology

## Abstract

**Background:**

The global poverty profile shows that Africa and Asia bear the highest burden of multidimensional child poverty. Child survival and development therefore depend on socioeconomic and environmental factors that surround a child.The aim of this paper is to measure multidimensional child poverty and underpin what drives it among children aged 5 to 18 years in a resource poor region of Burkina Faso.

**Methods:**

Using primary data collected from a cross sectional study of 722 households in the Mouhoun region of Burkina Faso, the Alkire–Foster methodology was applied to estimate and decompose child poverty among children aged 5–18 years. Seven broad dimensions guided by the child poverty literature, data availability and the country’s SDGs were used*.* A binary logistic regression model was applied to identify drivers of multidimensional child poverty in the region.

**Results:**

The highest prevalence of deprivations were recorded in water and sanitation (91%), information and leisure (89%) followed by education (83%). Interestingly, at *k* = 3 (the sum of weighted indicators that a child must be deprived to be considered multidimensionally poor), about 97% of children are deprived in at least three of the seven dimensions. At k = 4 to k = 6, between 88.7 and 30.9% of children were equally classified as suffering from multidimensional poverty. The odds of multidimensional poverty were reduced in children who belonged to households with a formally educated mother (OR = 0.49) or stable sources of income (OR = 0.31, OR = 0.33). The results equally revealed that being an adolescent (OR = 0.67), residing in the urban area of Boromo (OR = 0.13) and rural area of Safané (OR = 0.61) reduced the odds of child poverty. On the other hand, child poverty was highest among children from the rural area of Yé (OR = 2.74), polygamous households (OR = 1.47, OR = 5.57 and OR = 1.96), households with an adult head suffering from a longstanding illness (OR = 1.61), households with debts (OR = 1.01) and households with above five number of children/woman (OR = 1.49).

**Conclusion:**

Child poverty is best determined by using a multidimensional approach that involves an interplay of indicators and dimensions, bearing in mind its causation.

## Background

In recent years, child poverty has taken the centre stage in global debates in both industrialised and developing countries. According to the World Bank [1] estimates, out of 767 million people living in extreme monetary poverty, 385 million of them were children below 18 years. The Human Development Index (HDI) also estimates that out of 1.5 billion people considered multidimensionally poor, 750 million were children. Although overall extreme poverty levels have declined by three times from 2.2 billion in 1970 to 705 million in 2015 [2], this phenomenon is unequally distributed across Africa, where nine out of ten children in Sub-Saharan Africa (SSA) live under some form of poverty [3]. This reiterates the certainty that poverty assessment in children is an important subject that cannot be overlooked or neglected.

There has been a great discord among researchers and policy makers on how best to measure and define poverty. In the monetary approach, poverty can be considered as absolute, relative or in terms of a poverty line [4]. The former defines children as poor if the household income is unable to uphold certain basic living standards or afford a certain basket of goods and services [5]. Relative poverty on the other hand is when the household income is below a certain threshold of median income in the country. Using the poverty line, families are considered poor if the family income fall below the international poverty line commonly used as 1.9$ per day [6]. Critics hold that this unidimensional approach does not take into consideration specific needs of the child at various age groups [7] and it underestimates poverty given that it does not capture intra-household resources [8], where some children or household members work and increase the household income. Child poverty therefore goes beyond this money-metric approach to mean the absence of basic social amenities like adequate housing, availability of clean drinking water and sanitation facilities, access to good health, adequate nutrition, education, information and leisure etc. Bearing this in mind, the United Nations Convention on the right of the child reinforces the need for children to enjoy the highest level of health and adequate living standard [9]. Therefore, through welfare dimensions and indicators, the general standpoint of using a multi-dimensional approach in measuring child poverty is widely acceptable.

Child poverty is a phenomenon largely experienced in the sub-Saharan region where over 67% of children in 30 countries are multidimensionally poor [10]. In Uganda for example, over half of the population are children under 18 years of age with 57% living in multidimensional poverty [11]. Following a war dispute and subsequent oil shut down in South Sudan, the country is plagued with income poverty, deprivations in the aspects of health, nutrition and education [12]. The highest incidence of poverty in Nigeria is in the Northern regions owing to Boko Haram insurgency where children lack homes and live in camps with poor access to food and health services [13]. The impact of poverty is most devastating on children. Children from poor households often suffer from social exclusion, low birth weights, mortality, poor health outcomes in adulthood and more likely to remain poor throughout life [14–16]. It is rather unfortunate that Africa endowed with its own natural resources still contains the largest number of poor persons in the world. Corruption, lack of good governance and policies are responsible for some of these societal drawbacks.

Burkina Faso is a West African country with a population of about 18 million inhabitants [17]. A study conducted by the Oxford Poverty and Human Development Initiative (OPHI) in 2010 indicated that 81% of the population live below poverty lines and 83% of the national population are multidimensionally poor [18]. The most deprived dimensions include living standards where over 80% are deprived in electricity, 78% in sanitation, followed by education and health. These statistics are quite high and of concern particularly in this period where the topmost goal of the Sustainable Development Goals (SDGs) is to end poverty by 2030. However, it is worth noting here that child mortality is reducing in the country given the various interventions at different levels of the health system. Rather, few interventions exist in relation to the other dimensions of living standards hence the high incidence noted.

In the education sector, adult literacy rate in Burkina Faso is as low as 28.6% and net enrolment for primary school is 63.2% [19]. Also, primary school completion rate in the country stands at 61.6%. School attendance is worst in the rural setting especially during the rainy seasons where families engage in agricultural activities for income generation. The poor road infrastructure in rural communities in Burkina Faso, the absence of affordable and organized transport system and hence long walks to school, coupled with low quality of teachers all impede a child’s education and school performance [17]. Older children often drop out from school, migrate to the towns in search of work to improve the family’s impoverished status. Education is undoubtedly a major drawback in mitigating poverty in the region. In terms of nutrition, over 954,000 people in the country experience food insecurity and 133,000 children under five suffer from severe malnutrition [20]. The frequent droughts, floods and other harsh weather conditions affect agricultural productivity and subsequent nutritional status of the indigenes. Generally speaking, the high illiteracy rates, high unemployment rate and low-income levels in the country worsen the poverty rate of Burkinabés likewise their nutritional development. Families face hardships given the poor economic situation in the country to make ends meet. Children bear the burden of family misfortune such that a vicious cycle of poverty continues to adulthood or lead to death before the age of 5 years.

Despite the country’s stable economic growth over the past 6 years, poverty levels keep rising especially among children. This is to say that in capturing child poverty, it is imperative to explore other aspects of poverty as well as identify poverty drivers in order to build interventions in relation to findings. Findings from literature show that child poverty can be associated to parental neglect, religious beliefs, home violence, age of parent, wars/conflicts, famine, low family-household income, house size, migration as causative factors [21]. This study uses children aged 5 to 18 years because they are often given less attention in most intervention programs. Furthermore, few studies exist on child poverty measurement in Burkina Faso. Most poverty studies in the country focus on the general population [18] and others measure poverty using the money metric approach.

This paper therefore uses a multidimensional approach in measuring child poverty, focusing on the vulnerable age group, children and adolescents because they remain dependent on their parents for support and survival. Secondly, given that child poverty is a backlash to societal growth and development and hinders talented young minds to make an impact in their own society, the outcome of this study will be used to shape policies and interventions that favour child well-being in general to mitigate the effect of child poverty in adulthood. Though the money metric approach to poverty has made significant contribution in understanding child poverty, it is however insufficient and not representative of child wellbeing in multiple dimensions that are equally important to children rights such as health, nutrition, education, shelter, sanitation among others. When a child’s wellbeing is viewed through a multidimensional *lens of the child as an individual and then through societal and institutional conditions surrounding* his birth, growth and living conditions, then a label can be attached to the child as either poor or not [22]. Our study seeks out to measure child poverty using the Alkire and Foster’s multidimensional approach [23] and explore what drives multidimensional poverty in this age group.

## Methods

### Study area and data

Located in the northwest of Burkina Faso, the Boucle du Mouhoun region (12°30′N; 3°30′W) whose headquarter is Dédougou, occupies about 12.6% of the national territory (about 34,333 km2). It encompasses approximately 10% of the total population of the country with a density of 53 inhabitants per square kilometre [24]. The country is bounded by the Republic of Mali, Ghana, Niger, Togo and Cote d’Ivoire.

Boucle du Mouhoun region was selected for several reasons including (i) the high prevalence of poverty (one of the poorest region in the country); (ii) higher proportion of young people under the ages 15 years (49.9%) and 25 years (68.1%); (iii) the negative migration balance: the region is a ‘hot spot’ for intra and inter rural/urban migration in the country with far reaching implications for national development.

**Fig. 1:**
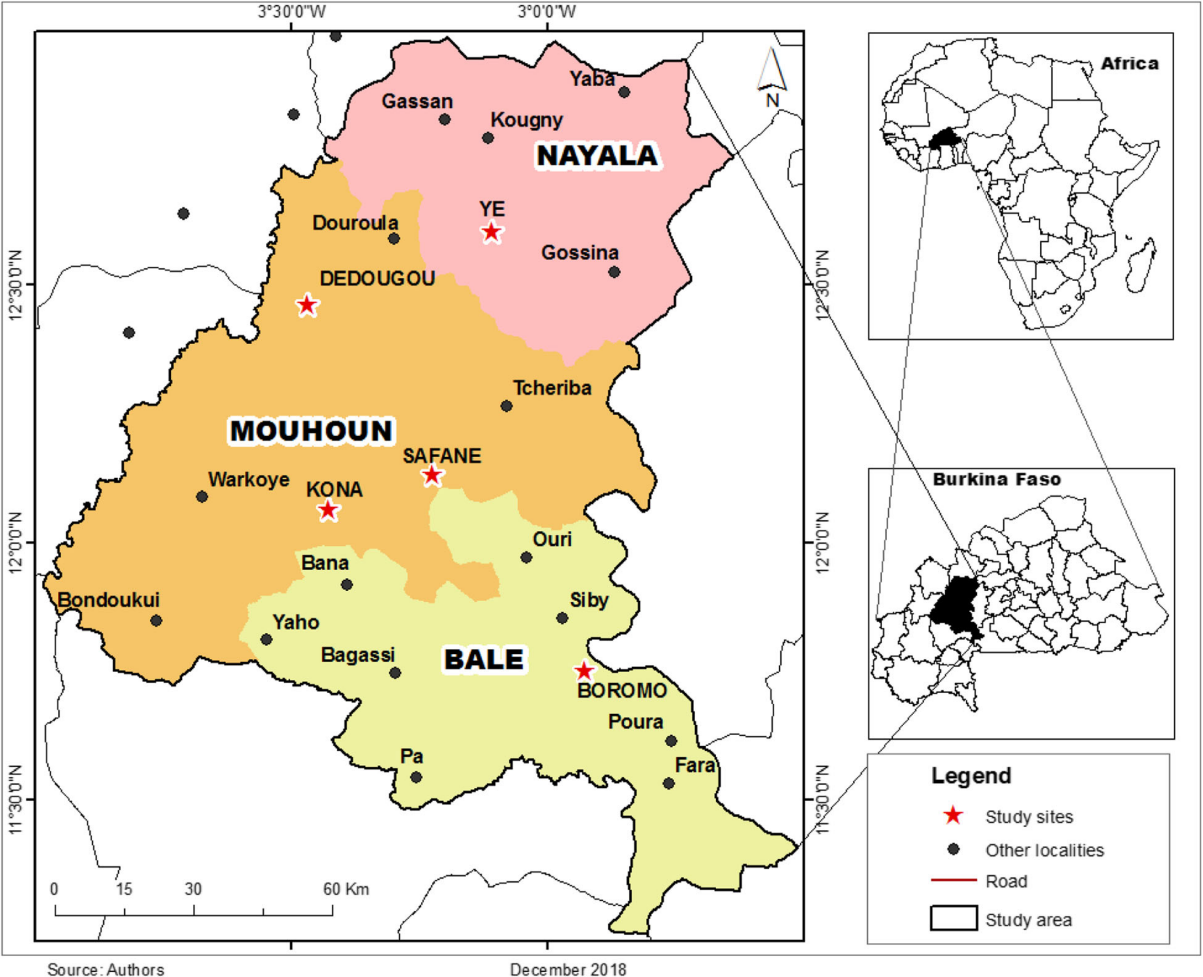
Overview of the Study Area. The map was created by the authors showing the Boucle du Mouhoun region and its three provinces, Nayala, Mouhoun and Bale. The survey was conducted in the two urban areas of Boromo and Dédougou, and three rural areas of Kona, Safané and Yé

The study used primary data from a UNICEF-Save the Children sponsored project on child poverty profiling and vulnerability in Burkina Faso. Five communities, including two urban communities (Dédougou and Boromo), and three rural communities (Safané, Kona and Yé) were purposively selected. These communities are located in three provinces (Balé, Mouhoun and Nayala) of the Boucle du Mouhoun region (Fig. 1). The five communities were selected on the basis of high poverty incidence as recommended by the office of UNICEF Ouagadougou. A total of 20 enumerated areas (EAs) were randomly selected from all 5 communities. Given that 60% of the population of the region were from rural areas, 12 out of 20 EAs were randomly selected from Safané (6), Kona (2) and Yé (4), while 8 EAs were selected from the urban areas of Dédougou (6) and Boromo (2).

The required primary sampling units for the EAs were numbered, and households were then randomly selected. The sample size calculation was based on the number of children including teenagers in the region, making a total of approximately 81,818 children. Applying the Taro Yamane formula [25] with a 5% margin of error, the minimum sample required for the study was calculated as follows:

$$ n=N/\left[1+N\left({e}^2\right)\right] $$

Where *n* is the sample size to be estimated, *N* is the population size and *e* is the error margin (*e* = 0.05).Based on this specification, we obtained a minimum sample size of about 794 children aged 0–18 years. We avoided the error in non-response by adjusting the sample size by 20%. This resulted to a sample size of 952 respondents, approximated to 1000 respondents aged 0–18 years. However, the inclusion criteria for this study involved children aged 5 to 18 years, which reduced the sample size to 722 children.

The survey used a structured interviewer administered questionnaire divided into three parts; a section for household characteristics, children’s characteristics and mother’s characteristics. The household heads were directed to the sections specified for household characteristics. The mothers responded to their specific sections and the children’s section if the child was below 10 years of age. Adolescents responded to the children’s section with occasional interventions from the mothers when needed. The questions in the study tool were adopted from developing countries National Living Standard Survey Measurements (NLSS), OPHI modules, ‘Bristol Approach’ by UNICEF, Multiple Indicator Cluster Surveys (MICs) by Alkire and Foster including other national surveys in Burkina Faso. The questions were equally adapted to suit the content of the study.

Data quality was ensured by doing a pilot study to test the survey instruments and identify potential errors for corrections. There were in total 11 trained graduates as enumerators and 3 field supervisors. The field supervisors in the beginning conducted 4 interviews per day with the enumerators to monitor their progress and check for data inconsistencies. Data entry was simultaneously done alongside data collection in case errors were identified. Digitalizing the data minimized error risks during data processing, i.e. entering the correct codes for the responses. This was done using data capture mask designed with Census and Survey Processing System (CSPro) software package version 5.0. Data was cleaned and analysed using SPSS (IBM SPSS Statistics for Windows, Version 20.0) and STATA 13.

#### Choosing dimensions and indicators

Measuring multidimensional child poverty and deprivation requires the identification of relevant dimensions in relation to public ideals. In this study, seven broad dimensions were identified for the multidimensional frame work as shown in Table 1. The selected dimensions were specifically chosen to capture progress in the country’s MDGs. These include nutrition, health, education, water and sanitation, housing, information and material deprivation (per capital income). Each of the dimensions were measured using well-defined indicators drawn from the literature on child poverty [26]. Note that each indicator was assigned equal weights assuming that each counts equally in a child’s wellbeing and development in the society as suggested by the Convention on the Rights of the Child [9].

**Table 1:** Dimensions specific deprivation cutoffs and weights for children aged 5–18 years

Dimension	Deprivation Cut-off	Indicators	Weight
Household Related			
Housing	Children living in a house with 4 or more persons per room (overcrowding), inadequate floor (ground/plank), and wall materials (mud/clay/thatched, or living in a household with no access to any form of electricity.	4	1/7%
Water and Sanitation	Children are considered deprived under these dimensions; if they use unprotected well/rain water or river/stream/ lake /pond as main water source and have no toilet facilities or share toilet, use unimproved pit latrines or practice open defecation.	2	1/7%
Household Income	Children leaving in households that fall in the last two quintiles of household per capita income distribution	1	1/7%
Information	Children are considered deprived under this dimension if they lack access to communication and media broadcast.	2	1/7%
Child Related			
Nutrition	Children are deprived under this dimension if their BMI-for-Age or Weight-for-Age was −2 standard deviation below the reference mean	2	1/7%
Health	Children are considered deprived in health if they did not get healthcare when needed or from a household with an incidence of child mortality.	2	1/7%
Education	Child is not enrolled in a school, or was enrolled lately in school (age 7 years and above), doesn’t go to school daily, or a drops out from school.	4	1/7%

Family income is used as a dimension because a stable income provides family security and influences child development and growth [27, 28]. In using multiple dimensions to define child poverty, it is important to include the dimension, income that offers command over non-market goods [29]. Children from low-income households in Burkina Faso run the risk of engaging in child labour activities like mining, hawking to supplement the family budget. By so doing, they are often exposed to the risk of unwanted pregnancy, juvenile delinquent behaviours and poor school attendance increasing the tendency to drop out of school. In this analysis, children are considered deprived in income if they come from households that fall within the last two quintiles of household per capital income distribution.

A second dimension is housing. A child’s dwelling can affect his psychosocial well-being as well as expose him to certain health risks [30]. Burkina Faso is a country with very harsh weather conditions especially during the rainy seasons where houses are often flooded with water and debris, increasing the risks of infectious disease spread. Individuals may lose poorly built homes to strong winds and flooding, putting the family at risk of migrating and squatting from one home to another. Additionally, electricity is an indicator included in this dimension not only because it offers some form of family satisfaction but rather a booster to a child’s performance at school. A child is considered deprived in housing if he or she lives in a house without electricity, or the house is not made of formal roofing or wall construction materials, or sleeping in an overcrowded house (i.e., 4 or more persons per room). Person’s per room is a measure of the indicator overcrowding and has been a subject of debate for over a decade [31]. Some scholars refer to it as an objective variable that must take into consideration the age difference of occupants in the room, the space and size of the room. What others see as overcrowding may not necessarily be overcrowding in another context. This study uses the UN definition and other previous studies on child poverty to define overcrowding. That is, 4 or more persons living in a tiny room thereby increasing the risk of infectious disease spread and violence [32, 33].

The dimension water and sanitation include provision of clean drinking water and availability of improved toilet facilities. These are the most basic and cost-effective ways of improving health in impoverished communities. Children are deprived in water and sanitation if they use unprotected well/rainwater or river/stream/ lake /pond as main water source and have no toilet facilities or share toilet, use unimproved pit latrines or practice open defecation.

We used the Composite Index of Anthropometry Failure (CIAF) to assess the nutritional status of children by forming a composite nutritional index, Under-nutrition [34]. For older children (5–9 years), the recommended nutritional assessment is BMI-for-Age (BAZ), an indicator for Wasting or Thinness and Height-for-Age (HAZ), an indicator for Stunting [35]. The World Health Organization’s Anthoscore software was downloaded and used to construct the indicators for Wasting and Stunting. A child is defined as suffering from Wasting or Stunting if he falls − 2 standard deviations (SD) below the referenced population mean. A child was suffering from Undernutrition if he was either wasted or stunted or suffering from both (Table 2).

**Table 2:** Composite index for anthropometry failure (CIAF)

Description	Wasting	Stunting
No failure	No	No
Wasting only	Yes	No
Stunting only	No	Yes
Wasting and Stunting	Yes	Yes
Total		
CFIA = Under-nutrition		

To reduce maternal and child mortality rates in the country, a free health initiative for pregnant women and children under-5 years of age was implemented in the country in 2015/2016. Prior to this, the Integrated Community Case Management (iCCM) of childhood illnesses has been a strategy implemented at community level to provide healthcare services in hard to reach areas. This intervention aimed at improving access to healthcare services and thus improve child survival. However, it mainly focuses on children under 5 years while little is known about health care access for older children. It is therefore interesting to determine the extent of healthcare access among older children who rely on out of pocket payment for medical expenses. A child is considered deprived in health if he or she did not get healthcare when last needed or if the child is from a household with an incidence of child mortality.

With regards to information, children need the media to improve on their intellectual capacity as well as shape certain behavioural norms. It is essential for children to live in households with access to phones especially for school emergencies. A child is thus, classified as deprived in information if he or she lives in a household without radio or television or from a household the lacks access to a mobile phone.

The importance of child education cannot be overemphasized as it improves an individual’s social status and standard of living later in life. It is not enough to enrol a child in school but equally important to monitor school frequency and school dropout among this vulnerable age group. A child is considered deprived in education, if he or she is a school drop-out, or was not enrolled in a school, or was enrolled in school late (age 7 years and above) or does not go to school daily.

#### Computing multidimensional poverty index (MPI)

In this section, we calculate the multidimensional poverty index (MPI). The first question asked is, who are the poor? Bourguignon and Chakravarty [36] identified the poor as those deprived in *any of the dimensions* being explored. While this is a useful place to start, it does not look *across dimensions* to label individuals as poor. Alkire and Foster (AF) [23] use a more practical approach in measuring multidimensional poverty, which takes into consideration the number of dimensions an individual is deprived. The two methods of identifying the poor include the union and the intersection approach. In the union approach, an individual is considered poor if deprived in *at least one* dimension. This is theoretically intuitive but practically improbable because almost everyone will be considered poor if studying a large population. Thus, it represents a bias of inclusion [37]. The latter on the other hand considers a person as poor if deprived in *all dimensions*. Again, this method fails to identify persons who are deprived in certain dimensions and not in the other. For instance, a healthy child may not be considered poor if he did not go to school or lives in a low-income household. There is therefore the tendency to underestimate poverty.

These two approaches are balanced in the AF’s dual cut-off approach, which builds on Sen’s two basic principle namely; identifying the poor and constructing an index to determine the extent of poverty [38]. As the name implies, two cut-offs are established to define multidimensional poverty. First is the deprivation cut-offs, that determines if a person is deprived in any of the dimensions and then the poverty cut-off which determines how extensively deprived a person should be to be considered poor [39]. The AF’s methodology goes through a series of steps namely, defining the indicators used, setting the level of deprivation cut-offs for each indicator, assigning equal or differential weights to the indicators and summing each up to one, ascertaining if an individual is deprived or not, creating a weighted sum of deprivations for everyone and lastly determining the poverty cut-off that identifies an individual as multidimensionally poor. This phase is also known as the identification phase. The next phase, aggregation phase, calculates the following; the head count ratio (H_O_) which identifies the proportion of individuals who are multidimensionally poor, the intensity of multidimensional poverty (A) defined as the average share of weighted indicators in which poor children are deprived in, the adjusted head ratio (M_O_) calculated as product of the head count ratio and intensity of multidimensional poverty (HxA).

To compute the multidimensional measures, the paper uses the cut-off value *k,* which by definition, is the sum of weighted indicators that a child must be deprived in order to be considered multidimensionally poor [40]. It is equally seen as a policy variable describing the range of deprivations each poor child must have to be classified as being deprived. Following Alkire and Santos definition, a child is considered multidimensionally poor if the weighted indicator (*k*) of which he or she is deprived is greater than or equal to 33.3% [41]. In this paper, we differentiate three broad categories of poverty based on similar precepts. That is; *The Non-Poor Children* (*k* = 1), *Children who are Vulnerable to Poverty* (*k* = 2), and *Multidimensionally Poor Children (k ≥* 3) [42].

#### Logistic regression estimates

We identify factors associated with *Multidimensional Poverty* using binary logistic regression models at 5% level of significance. The dependent variables for the binary models are poverty/deprivation used in computing the headcount (H_O_) for each of the poverty construct (*k =* 3*, k* = 4, *k* = 5 and *k* = 6). Four models were used to obtain a comprehensive picture of drivers of child poverty. A deprived child has a value of '1' while a child who is not deprived has a value of '0'. Some of the predictors explored in this analysis include the age of household head, adult health, child age, area of residence, household size, education status of household head, marital status and household debts status among others. It is hypothesized from the literature that these household characteristics were associated with child poverty. The explanatory variables were measured thus:

Adult health and mother’s health status were defined as those diagnosed with longstanding illness in the past 12 months. The conditions assessed were Diabetes, Asthma, Low Back Pain, Hypertension, Angina, Depression, Arthritis, Chronic Obstructive Pulmonary Disease, Cancer and Others to specify. Those in the ‘others category’ with HIV and other long-term ill-health from unknown cause were included in the yes group. Adults and mothers with longstanding illness were coded '1' meaning ‘Present’ and those without as '0' meaning ‘Absent.’

An indebted household was defined as households where the household head or other members of the household were in debt. This measure was included because previous studies show that households with unmanageable debts have higher chances of compromising a child’s general well-being [43, 44].

Given that over 80% of the indigenes in the region were engaged in agriculture, income sources were categorized into three categories. Those who had never worked or having any source of income what so ever (no income), income derived from non-farm activities in both private and public sectors including transfers (non-farm incomes), and incomes from agricultural activities (farm incomes).

Adult education status was grouped into no formal education, formal education (primary, secondary or tertiary school attendance) and informal education (koranic or adult education).

A household size of below eight members was considered normal in the African setting where nuclear families often live with extended family members. In Burkina, the average rural household size is normally 8 persons [45].

Despite global decline in fertility rates, SSA still experience a slow decline in fertility rates. The average woman in SSA desire to have 4 to 5 children [46]. It is on this precept that number of children per woman was coded ‘0’ if the number of children/woman was between 1 and 5 and ‘1’ if 6 and above.

## Results and discussion

### Results

Table 3 presents the socioeconomic and demographic characteristics of children aged 5–18 years in the sample. It comprises of two groups: pre-adolescents (5 to 9 years), which constitute 39% of the total sample, and 62% of adolescents aged 10 to 18 years. The results showed that 52% of the children were boys. Majority of children (59%) resided in the rural areas. More than 47% of children lived in homes where the mothers had between one to five children while 53% lived in homes with over five children/woman. In terms of education status, 53% of children within this age group lived in homes where the household head had no formal education and just about 16% came from households with a formally educated household head.

**Table 3:** Descriptive summary of children aged 5–18 years

Variable	Frequency (N)	Percentage (%)
Age group of children		
Pre adolescents (5–9)	278	38.5
Adolescents (10–18)	44 4	61.5
Sex		
Male	376	52.1
Female	346	47.9
Locality		
Urban	300	41.6
Rural	422	58.5
Number of children/woman		
One to five	388	53.7
Above five	334	46.3
Age group of household head		
Youthful age (22–35) years	98	13.6
Middle age (36 59) years	480	66.5
Old age (60 and above) years	144	19.9
Education status of household head		
No formal education	385	53.4
Formal education	118	16.4
Informal education	218	30.2
Longstanding illness of household head		
Absent	601	83.2
Present	121	16.8
Marital status of household head		
Monogamy	408	56.5
Polygamy	293	40.6
Single	21	2.9
Household size		
Less than eight persons	321	44.5
Eight and above persons	401	55.5
Mother’s education		
No formal education	586	81.2
Formal education	84	11.6
Informal education	52	7.2
Longstanding Illness of Mother		
Absent	412	57.1
Present	310	42.9
Household Income		
Lower	467	64.7
Middle	173	24.0
High	82	11.3
Household debt status		
No debt	270	37.4
In Debt	452	62.6
Religious denomination		
Christian	115	15.9
Protestant	42	5.8
Muslim	488	67.6
Traditional	77	10.7
School enrolment of child		
Not enrolled	299	41.4
Enrolled	423	58.6
Reasons for not attending school/dropping out		
Financial Problems	23	3.2
Health reasons	7	1.0
Distance to school	4	0.6
Work	5	0.7
Given to marriage/pregnant	66	9.1
Parent’s wish	42	5.8
Personal wish	126	17.5
Other	22	3.1
Not applicable	299	41.4

Most children (57%) were from monogamous families, 40% were from polygamous homes, and just a few proportion (3%) were from single-parent homes. Most of the household heads (67%) were of middle age (36–59 years). Over 44% of the children were from households with at least seven household members while 56% of children came from households with more than seven members. In relation to the mother’s education status, the results indicated that an overwhelming majority of mothers (81%) were illiterates; and 57% of children were from homes where the mothers did not suffer from any of the following longstanding illness (asthma, low back pain, hypertension, diabetes, chronic obstructive lung disease and depression).

The results also showed that most children in the study area lived in very poor and indebted households. As an illustration, approximately 65% of children were from households that fall within the low- and lowest-income categories, and about 63% children lived in households with debts. Religion wise, majority of the children 68% of children came from Muslim homes compared to 16% of children from Christian homes. While about 59% of children were enrolled in school, 41% were not yet enrolled. The highest reason for not attending school or dropping out from school were personal (17%), followed by early marriage (9%) and then parent’s wish (6%).

### Prevalence of deprivation

The results in Table 4 show the percentage of children classified as poor in each specific indicator and dimension and not necessary being multidimensionally poor. It is important in that it helps in sector specific poverty targeting. As observed, on average, the prevalence of deprivation among children in the region is highest in the area of water and sanitation (91%) followed closely by the dimension information and leisure (89%). Concerning water and sanitation, this is largely attributed to poor sources of drinking water and lack of access to improved toilet facilities. In fact, the analysis reveals that over 90% of households in the region use unprotected well, rain water and river, stream, lake, or pond water as the main source of drinking water. Similarly, it was observed that more than 92% of the households practice open defecation, have no toilet, use uncovered pit latrine, pail or bucket or share toilet facilities. This explains why the prevalence of deprivation among children in the region is highest in the dimension water and sanitation.

**Table 4:** Distribution ofchild deprivation by sex and region

	Income Quintile	Housing	Water & Sanitation	Nutrition	Health	Information & Leisure	Education
All	72.7	67.7	91.3	22.7	61.8	89.1	83.5
Sex							
Male	72.3	67.2	92.8	24.47	63.6	84.6	83.5
Female	73.1	68.2	89.6	20.81	59.8	93.9	88.5
Pearson’s x^2^	0.814	0.791	0.125	0.241	0.302	0	0.996
Districts							
Boromo	68.8	67.5	79.2	23.8	45.5	88.3	76.6
Dédougou	79.8	71.7	98.21	23.4	52.5	82.5	90.1
Kona	65.3	52.0	92.0	21.33	73.3	93.3	72.0
Safané	69.9	56.6	89	19.14	67.5	92.3	86.6
Yé	71.7	86.9	89.9	26.81	71	92.8	78.3
Pearson’s x^2^	0.055	0.000	0.000	0.544	0.000	0.004	0.000
Locality							
Urban	77	70.7	93.3	22.04	50.7	84	86.7
Rural	69.7	65.6	89.8	23.67	69.7	92.7	81.3
Pearson’s x^2^	0.029	0.051	0.098	0.607	0.000	0.000	0.055

The prevalence of deprivation is also higher for education (84%), where children suffer a lot from late age of enrolment (82%), poor school attendance (43%) or no enrolment/dropouts (41%). Other dimensions such as household per capital income, health and housing show moderate levels of deprivation. Specifically, 72% of children come from households that are deprived in income, 67% from households that are deprived in housing, and 61% from households that are deprived in health. Nutrition on the other hand, had the lowest prevalence rate of 22.1% among children.

Interestingly, when the prevalence of deprivation is disaggregated by sex, we found no significant gender differences in deprivation for all the dimensions except in information and leisure, where female children tend to be more deprived than their male counterparts. Disaggregating by districts shows that on average, deprivation is highest in water and sanitation (98%) followed by Information and leisure (93%). This occurs in the districts of Dédougou and Kona respectively. By locality (rural vs. urban), deprivation in the rural areas is significantly more pronounced in information and leisure (93%), and health (70%). In the urban areas, the highest deprivation was significantly seen in income (77%). These associations were set at 5% level of significance. The implications of these findings are taken up in the discussion section.

### MPI decomposable results

The prevalence of deprivation though very informative in the sense that it gives a broader picture of the proportion of children deprived in each dimension, it does not provide in-depth information on aggregate poverty measures. These measures are considered collectively useful for aggregate poverty reduction strategies. They include the headcount ratio (H_O_), the multidimensional adjusted Headcount ratio (Mo), the intensity of deprivation (A) and the average deprivation intensity among the deprived children. Table 5 presents the poverty measures using the AF method for different poverty cutoff values (*k*). In other words, *k* represents a range of deprivations a poor child must have to be considered deprived.

**Table 5:** Multidimensional child poverty measures for all *k* (5–18 Years)

Deprivation Threshold (*k*)	Multidimensional Headcount Ratio [*H*]	Adjusted Headcount Ratio [*M*o = *H* x A]	Intensity of Deprivation [*A*]	Average Deprivation Intensity among the Deprived [*A*(*k*) = *A* x *D*]
1	100	70.0	0.70	4.8
2	99.1	69.7	0.703	4.9
3	97.0	69.1	0.712	4.9
4	88.7	65.0	0.738	5.2
5	60.0	53.1	0.792	5.5
6	30.9	27.0	0.884	6.1
7	5.80	5.8	1.000	7.0

As observed in Table 5, it is interesting to note that all poverty measures (H_O_, Mo and A) decrease as *k* increases. That is, as the number of deprivations children suffer from increases, the number of children in poverty reduces. For instance, when the poverty cutoff value is set at one (*k* = 1, which corresponds to Alkire’s union approach), 100% of the children are deprived in at least one of the dimension used in the study. As *k* is increased, more and more children come out of poverty. For example, setting *k* at 7 (Alkire’s intersection approach), only about 5.8% of children are considered poor or deprived in all seven dimensions. It is important to also note that as the poverty cutoff value increases (fewer children in poverty), the intensity of deprivation as well as the average deprivation intensity among the deprived children increases. This semblance has policy implications in that poorer children are more represented when the cut-off is below 50%.

For policy purposes, we defined and used one broad classification of MPI in this paper as used in the literature. That is, *k* ≥ 30% or *k* ≥ 3, which by definition, is broad enough to include children that are deprived, irrespective of the number of dimensions they suffer from. The advantage of using this approach is that it provides a wide range of policy options useful for aggregate poverty targeting, thus offering policymakers with a wide array of choices and tradeoffs in designing child-sensitive poverty programs. For example, when *k* is set at 3, over 97% of children in the region are considered *Multidimensionally Poor* and on average, are deprived in at least 4.9 dimensions. This also corresponds to a poverty intensity of about 71%. On the other hand, if we set *k* at 4, though only 88.7% of children are now classified as *Multidimensionally Poor*, the average deprivation intensity among these deprived children increases to 5.2 with a poverty intensity of 73%. The same can observed when *k* = 5. While the multidimensional headcount ratio reduces to 60%, the intensity of deprivation increases to 79% and on average, poor children are deprived in more than 5.5 dimensions. This invariably shows the importance of *k* as a critical policy variable in programs that target child poverty. Thus, if the essential goal of the child-sensitive poverty targeting program is to improve children’s wellbeing irrespective of the number of beneficiaries, then increasing the cut-off value of *k* is highly desirable. On the other hand, if the goal of the child-sensitive poverty program is targeted to more deprived children and fewer indicators and dimensions, then reducing the poverty cut-off value would be advantageous [47].

**Fig. 2:**
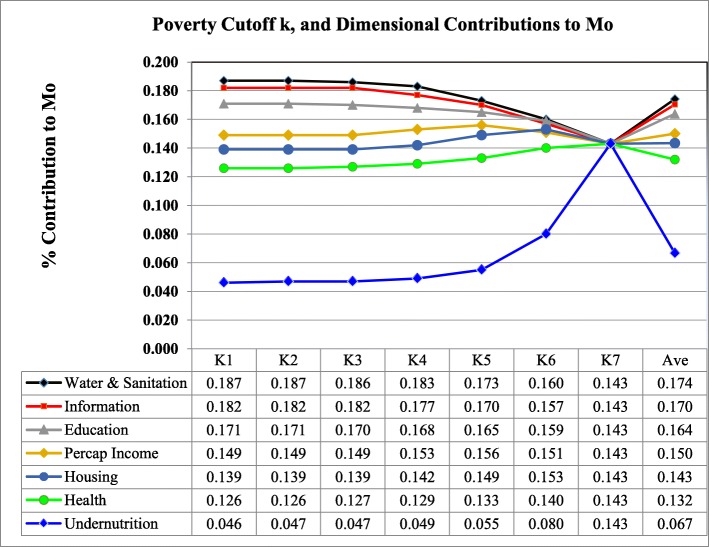
Dimensional Contribution to Mo. The dimensions Nutrition, Water and Sanitation and Education contribute highest to the adjusted multidimensional head count ratio, Mo. This visual representation of dimensional contributions to multidimensional poverty is quite helpful for policy targeting purposes in prioritizing interventions

However, because policy makers are often interested in targeting specific groups of the poor, it is crucial to decompose the dimensional contributions to child poverty indices at different cut-off values as shown in Fig. 2. In other words, Fig. 2 simply illustrate which of the dimensions contribute the most to child poverty measures in the region for the different poverty cutoff values used. As observed (Fig. 2), on average, the dimensions water and sanitation (0.174), information and leisure (0.170), education (0.164) and household per capital income (0.150) contribute the most to child poverty measures in the region. This invariably implies that, beyond the basic raw headcount statistics, aggregate poverty measures provide very useful information for precise poverty targeting purposes.

### Drivers of child poverty

In Table 6, we present the drivers of child deprivation and poverty in the region using binary logistic regression models fitted for a range of values of k ≥ 3. As observed, in model 1 (*k* = 3), this analysis revealed that that children from the urban community of Boromo were 87% (OR = 0.13, *P* < 0.05) less likely to suffer from multidimensional poverty. Children from polygamous homes were 5.6 times more likely to suffer from poverty (OR = 5.57, *P* < 0.05) compared to children from single homes. In model 2 corresponding to *k* = 4, the risk of poverty was 1.9 times higher in polygamous homes as well (OR = 1.93) and 3% higher in households with family debts (OR = 1.03). Further, the odds of poverty were 2.2 times higher in children from household with less than 8 members (OR = 2.23, *P* < 0.05). Children from families with non-farm generating income activities were 69% less likely to suffer from multidimensional poverty (OR = 0.31, *P* < 0.05). The results from Mode 3 with *k* = 5, shows that families with more than 5 children per woman (OR = 1.50) and those from polygamous homes (OR = 1.47) had a 1.5 chance of suffering from poor. In the same way, the odds of poverty were increased in households that had no sources of income by 54% (OR = 2.54, *P* < 0.05). On the other hand, children from households with a formally educated mother (OR = 0.49, *P* < 0.05) and with a source of income (OR = 0.33, *P* < 0.05) were 51 and 67% less likely to suffer from poverty respectively.

**Table 6:** A logistic regression showing predictors of severe poverty

Variable	*k* = 3			*k* = 4			*k* = 5			*k* = 6		
	Odds Ratio	T-Stat	*P*-Value	Odds Ratio	T-Stat	*P*-Value	Odds Ratio	T-Stat	*P*-Value	Odds Ratio	T-Stat	*P*-Value
Age of Household Head (HH)												
Youthful age (21–35) years	1.00			1.00			1.00			1.00		
Middle age (36–59) years	6.374	1.61	0.106	0.8	−0.58	0.564	0.944	−0.22	0.826	1.529	1.52	0.128
Old age (above 60 years)	0.616	−0.79	0.432	1.021	0.06	0.954	0.822	−0.86	0.392	0.925	−0.35	0.725
Child Age												
Preadolescent (5–9) years	1.00			1.00			1.00			1.00		
Adolescents (10–19) years	2.062	1.26	0.209	1.483	1.37	0.169	1.205	1.01	0.312	^**^0.667	**−2.13**	**0.033**
Child Gender												
Male	1.00			1.00			1.00					
Female	0.979	−0.04	0.966	1.379	1.24	0.217	1.262	1.38	0.168	0.835	−1.05	0.295
Communities												
Dédougou												
Boromo	^**^0.133	**−2.5**	**0.012**	0.345	0.14	− 255	0.576	−1.79	0.073	0.581	−1.61	0.106
Kona	0.309	−1.13	0.257	0.676	0.32	−0.83	0.62	−1.58	0.115	0.746	−0.88	0.377
Safané	0.279	−1.56	0.118	0.898	0.33	−0.29	0.671	−1.73	0.083	^**^0.608	**−2.08**	**0.037**
Yé	0.298	−1.41	0.159	1.098	0.46	0.22	1.442	1.39	0.164	^**^2.253	**3.23**	**0.001**
Number of children/woman												
One to five												
Above five	1.602	0.84	0.402	1.284	0.88	0.38	^**^1.495	**2.18**	**0.029**	1.233	1.13	0.259
Education status of HH												
No education	0.423	−1.52	0.129	0.619	−1.34	0.181	1.021	0.08	0.934	0.769	−1	0.319
Formal education	2.585	1.34	0.18	0.615	−1.55	0.121	0.937	−0.32	0.751	1.14	0.63	0.529
Informal												
Longstanding illness of HH												
Absent												
Present	1.796	0.82	0.412	0.8742	−0.39	0.697	1.324	1.18	0.237	^**^1.617	**2.16**	**0.03**
Marital status												
Monogamy	1.00			1.00			1.00			1.00		
Polygamy	^**^5.571	**2.69**	**0.007**	^**^1.964	**2.29**	**0.022**	^**^1.473	**1.97**	**0.049**	1.464	1.89	0.059
Single	1			1	(empty)		1.315	0.53	0.599	1.064	0.12	0.906
Household Size												
Eight and above	1.00			1.00			1.00			1.00		
One to seven persons	2.779	1.74	0.082	^**^2.281	**2.63**	**0.009**	1.398	1.67	0.094	1.237	1.03	0.304
Mother’s education status												
None	1.00			1.00			1.00			1.00		
Formal education	0.49	−1.07	0.284	0.619	−1.34	0.181	^**^0.494	**−2.69**	**0.007**	0.829	−0.65	0.518
Informal education	0.287	−1.79	0.074	0.615	−1.55	0.121	0.666	− 1.25	0.212	0.647	−1.19	0.236
Longstanding illness of mother												
Absent	1.00			1.00			1.00			1.00		
Present	0.72	−0.64	0.521	1.156	0.53	0.594	1.194	0.99	0.32	1.148	0.77	0.441
Income source												
Farm activities	1.00			1.00			1.00			1.00		
Non-farm activities	0.406	−1.58	0.114	^**^0.311	**−3.96**	**0.000**	^**^0.656	**−2.09**	**0.036**	0.683	−1.64	0.1
None	3.703	1.41	0.159	1.944	1.58	0.114	^**^2.541	**3.97**	**0.000**	^**^2.472	**4.09**	**0.000**
Household debts												
HH without debts	1.00			1.00			1.00			1.00		
HH with debts	0.896	−0.21	0.837	^**^1.013	**0.29**	**0.04**	1.286	1.33	0.184	1.032	0.17	0.866

*P* < 0.05, **indicate significant difference at 5%

Finally, model 4 (*k* = 6) shows that adolescents had a 33% (OR = 0.67, *P* < 0.05) reduced risk of suffering from poverty compared to pre-adolescents. In terms of place of residence, those in the rural area of Safané were 39% (OR = 0.61, *P* < 0.05) less likely to suffer from poverty whereas those in the rural area of Yé were 2.3 times more likely to be poor (OR = 2.25, *P* < 0.05). Children from families where a household head suffered from a longstanding illness were 1.6 times (OR = 1.62, *P* < 0.05) more likely to be poor. No family source of income increased the chances of child poverty by 47% (OR = 2.47, *P* < 0.05).

Summarily, significant drivers that increase multidimensional child poverty in the Mouhoun region of Burkina Faso include; polygamous households, increasing number of children per woman, household heads with long standing illness, no source of income and residing in rural Yé. Factors that reduced the risk of multidimensional poverty include; belonging to the adolescent age group, being a mother with formal education, having a source of income, household size above 7 members and residing in the urban area of Boromo or the rural area of Safane.

## Discussion

In recent years, socioeconomic inequality seems to be a societal pattern of occurrence. While some people live under good conditions with clean drinking water, good housing facilities, adequate nutrition and good access to healthcare. A lot more people still lack these necessities. Our study finds high levels of child deprivations in all dimensions particularly water and sanitation, information and leisure and education except in nutrition. The low prevalence of undernutrition among older children is quite understood given that the general prevalence of undernourishment has reduced in Burkina Faso from 25.6% in 2001 to 20.1% in 2017 [48]. A plausible reason could be more independence in eating habits giving the rising consumption of food from food vendors and high consumption of sweetened foods. This is in keeping with the current era of double burden of malnutrition spreading across Africa [49]. Also, community interventions for nutrition are well established in the country over the years.

Deprivations in water and sanitation are surprisingly higher in the urban areas as well as in the rural areas. One such explanation could be the influx of indigenes from rural to urban areas. Given the economic hardship in Burkina, local migrants in cities find themselves in slums and low-cost housing areas where standard of living is so low and to a certain extent affordable. These families are often invisible to urban authorities and met with poor sanitary conditions, lack clean drinking water and high risks of infectious disease spread [50]. Overall, the deprivation prevalence across dimensions provides an interesting basis for sectoral poverty policy targeting. For instance, the results suggest that while information and leisure is very gender-sensitive, large significant disparities exist between urban and rural children with respect to household per capital income, health and again information and leisure. This provides a very compelling reason to focus deprivation-reducing efforts for rural children in improving information and health. The same applies to urban children. The focus should be on improving alternative sources of household income which is an enabler for families to improve their housing quality, sanitation, health access as well as nutritional status.

The analysis finds high rates of deprivations in education in the region. This is rather not surprising since the literacy level in Burkina is as low as 22.7% for women and 36.7% for men. Though primary school gross enrolment rate in the country including adult primary education has increased from 42.7% in 2000 to 81.3% in 2013, primary school completion rate remains low at 59.1% [51]. The 2014 national education profile in Burkina shows that over 48% of children of primary school age are out of school [52]. This explains the high rates of deprivations in education were most children don’t attend school or drop out of school to do miniature jobs like mining, hawking and farming in rural areas to supplement family income. Lack of such basic commodities like education, health, nutrition according to the indigenes is what defines poverty [53].

The world has agreed to eradicate extreme poverty by 2030 through the guidance of the SDGs. Though the economic situation in the country has improved as indicated by a GDP of 4.5% in 2004 to 6.7% in 2017 [54], Burkina remains a poor country. Likewise, in the region, irrespective of the study region’s agricultural potential often referred to as the “food bucket” of the nation [55], poverty still remains as high as 92% in the region [56], near similar to our study findings, when *k =* 3. This reinforces the disturbing narrative that a lot is needed to bridge the poverty gap particularly especially within the age groups of children and adolescents in the country. The government of Burkina along with its development partners are doing a lot to reduce under 5 mortality rates through free health initiatives for mothers and babies, integrated community case management, nutritional interventions at community levels, hence the decline in proportion of deprived children in these dimensions (health and nutrition) when compared to the other dimensions. A lot is yet to be done in improving sanitation and availability of clean water particularly in rural areas and urban slums.

We find that children in the rural areas of Yé were more likely to suffer from multidimensional poverty compared to the urban children in Dédougou. Supportive findings from literature highlight this assertion of uneven geographical inequality in terms of distribution of economic resources [57, 58]. Living in rural areas for example, with low job opportunities and high concentration of poor individuals increases the tendency of a child growing up poor. However, a rural area like Safané with high agricultural potentials and high cotton growth in the region showed a reduced chance of poverty.

The study found a negative correlation between maternal education and multidimensional poverty. Similar results in a cross-country study in SSA showed that child mortality levels dropped by 65 points for educated mothers in Burkina Faso [59]. The role of education in wellbeing is most times associated with economic independence and thus the ability to fulfil family responsibilities [60, 61]. Further, the analysis shows that children from households where the household head had a steady income source had less chances of plummeting into poverty. Parental employment increases the economic status of the family and of course the ability to improve a child’s living standards [62]. In the United States for example, studies have shown that lack of investments in parental employment increases the chances of family poverty which impacts negatively on children [63]. Most of these children face academic difficulties, health and nutritional inadequacies as well as emotional dissatisfaction. The most perilous path among teenagers is the risk of delinquent behaviour such as hard drug use, prostitution, high rates of crimes and violence.

For children, the family is the first portal of entry into the society. Family instability has long and devastating effect on children throughout childhood. This study shows a positive effect of polygamy and child poverty. We found that children from polygamous homes were more likely to be poor. The non-nuclear family structure creates a possibility of child neglect which impacts negatively on his intellectual functioning [64–66]. Still on family structure, we found that small household size was positively associated with poverty among this age group of children. Our result is contrary to other studies that showed that increasing family size comprises child well-being, education and quality of care [67]. However, in keeping with this study which showed reduced chances of poverty with small household size, White and Massett argue that this relationship is possible when economies of scale is applied [68]. The idea is that private goods are shared among household members such that cost of expenditure is reduced as well as family poverty. Also, since agricultural activities are the main activities of the indigenes in the Mouhoun region, extended family members may provide larger work force thereby increasing the family’s livelihood. Further still, extended family members may assist in shouldering some of the family’s financial responsibilities [69]. This is particularly common in low income households.

The study finds a positive relationship between household debts and child poverty, in line with previous studies [70]. Research also shows that children living in households with a parent suffering from longstanding illness or disability are more likely to live in poverty [71, 72]. Long standing illness lowers work productivity and earnings thereby enhancing family poverty [73]. In the same vain, we found a direct relationship between increasing number of children/woman and poverty. Child poverty is enhanced when parental attention is reduced and sibling quest for public goods like education and health care services are increased particularly when they are at a dependent age [74]. Studies have also found that low test scores were associated with high number of children in the family [75, 76]. Raising a child elevates parental stress which gets worst with more children. The risk of maternal and child mortality is often associated with high parity.

Addressing child poverty will require a multi-sectoral intervention approach that will incorporate the dimensions water and sanitation, education, information, health etc. into one basket. Access to clean drinking water, well improved toilet facilities, education and improved income levels are all rudiments to good health and development in children [77, 78]. As one of the strategies to bring about community development, the government of Burkina embraced a decentralised system of governance. The approach was to rule out the top-down approach of reaching out to the population and establish a bottom – up strategy that will give autonomy to local authorities and community members to take care of the issues that are of concern in their communities. Despite the existence of decentralisation, poverty persists in the country and hence no intended outcomes were produced. Bado [79] explains some reasons for the failed governing system to be the following: persistence of top-down approach to development by the government and NGOs, centralisation of power in the capital cities, excessive reliance on donor funds, weak empowerment of local authorities, poor accountability system etc. In this regard, it is not an oversight to perform community poverty studies to understand the problems and priorities of poor communities. Government and policy makers will be best informed and advised on the extent of deprivations for prioritisation in designing and implementing interventions.

## Limitation

One of the study limitations is the fact that income used as a dimension does not usually reflect true household income as people tend to under or over report their income. Secondly, this study is based on a cross sectional design and so it is hard to establish a causal relationship between multidimensional poverty and its predictors. Thirdly, it was very challenging to select the most appropriate indicators to use for each dimension given the wide range of indicators available in the child poverty literature. However, based on a comprehensive literature review and thorough research on child poverty, most indicators were carefully chosen from previous work. Lastly, the study’s data was unable to distinguish between stock flows and inflows indicators to know how long families have been in poverty and identify those that have fallen out of poverty. However, it is hoped that subsequent studies on child poverty will pay important attention to this important limitations often associated with cross sectional poverty data used for poverty analysis.

## Conclusion

This study has measured child deprivations and poverty in one of the poorest regions in Burkina Faso, using the Alkire and Foster’s multidimensional approach. Seven broad dimensions and indicators that captured the country’s SDGs were used. High levels of deprivations were noted in all dimensions except in nutrition. The highest deprivations were in water and sanitation, information and leisure, education and per capita income. About 97% of children were suffering from multidimensional poverty at poverty cut-off of *k = 3.* The study also identified an interplay of contextual factors associated with child poverty. A positive association was noted among children from polygamous homes, households with no family income source, households with above five number of children per woman, household head with longstanding illness, small household size and residing in the rural area of Yé. Whereas negative associations with poverty include households with an educated mother, households with income sources either from farm or non-farm activities and residing in the urban area of Boromo and rural Safane.

Children bear the burden of poverty with devastating health and emotional consequences late in life. The negative impact of child poverty is overwhelming. The study’s goal is to inform government, donor agencies and other stakeholders to design child sensitive programs for poverty targeting. To start with, the government is recommended to address socioeconomic factors that affect poverty by empowering parents and care givers through job growth and employment opportunities. It will serve as a way of increasing family finances and of course the proficiency to improve living standards. For example, the ability to afford basic family needs like clean drinking water, adequate and quality foods and the ability to pay for education and health. In this regard, the government of Burkina must strive to align the dimensional deprivations with the country’s development agenda goals. It is a fundamental right of every individual, most importantly children to have at least a decent standard of living free from social exclusion.

Given that over 70% of families engage in farming, it is necessary for government to scale up agricultural interventions at community level. For instance, the provision of drought resistant seeds to small scale farmers as well as train farmers on new technological insights to improve yields. The impact should be to improve livelihood and social status and thus the ability to afford public goods. Efforts by the government should be geared towards improving clean water by constructing more boreholes in the communities. Sensitisation campaigns should be used to discourage open defaecation and seek community effort to improve and make available toilet facilities. The government should invest more in solar energy given the long periods of heat and dryness in the country. It may be expensive to install but in the long run, cheaper and always available. Media is a necessary tool for transmitting public health campaigns and other important messages that can benefit the family’s wellbeing. The availability of electricity will motivate households to possess radios and TVs for the purpose of information.

Educating every child through provision of affordable public schools and quality training centres for potential teachers are rudiments to improving the education sector. Providing access to quality and affordable health services and living in a clean environment should be a public health priority and evidence of societal development. Summarily, a holistic approach is needed by civil authorities and other stakeholders to address the multifaceted causes of child poverty. Interventions should be considered at the level of the household head, child and the mother’s level who is seen the most important caregiver. At family level, improving family income by providing employment opportunities as well as agricultural innovations is a good place to start. Educating every child is building a resilient society in future. Educating mothers will earn women the freedom of financial independence and thus the opportunity to maintain a child’s wellbeing.

## Data Availability

Data for this study can be obtained on request from the Principal Investigator (w.fonta@afdb.org).

## Abbreviations

CIAF: Composite Index of Anthropometry Failure
EA: Enumerated Areas
HDI: Human Development Index
Ho: Multidimensional Head Count Ratio
iCCM: Integrated Community Case Management
Mo: Multidimensional Adjusted Head Count Ration
MPI: Multidimensional Poverty Index
OPHI: Oxford Poverty and Human Development Initiative
SD: Standard Deviation
SDGs: Sustainable Development Goals
SES: Socioeconomic status
SSA: Sub-Saharan Africa
WHO: World Health Organization
